# Correcting bias in self-rated quality of life: an application of anchoring vignettes and ordinal regression models to better understand QoL differences across commuting modes

**DOI:** 10.1007/s11136-015-1090-8

**Published:** 2015-08-09

**Authors:** Melanie Crane, Chris Rissel, Stephen Greaves, Klaus Gebel

**Affiliations:** Prevention Research Collaboration, Sydney School of Public Health, University of Sydney, Sydney, NSW 2006 Australia; Institute of Transport and Logistics Studies, The University of Sydney Business School, University of Sydney, Sydney, NSW 2006 Australia; Centre for Chronic Disease Prevention, College of Public Health, Medical and Veterinary Sciences, James Cook University, Cairns, QLD 4870 Australia

**Keywords:** Quality of life, Differential item functioning, Anchoring vignettes, Commuting, Cycling, Ordinal logistic regression

## Abstract

**Purpose:**

Likert scales are frequently used in public health research, but are subject to scale perception bias. This study sought to explore scale perception bias in quality-of-life (QoL) self-assessment and assess its relationships with commuting mode in the Sydney Travel and Health Study.

**Methods:**

Multilevel ordinal logistic regression analysis was used to analyse the association between two global QoL items about overall QoL and health satisfaction, with usual travel mode to work or study. Anchoring vignettes were applied using parametric and simpler nonparametric methods to detect and adjust for differences in reporting behaviour across age, sex, education, and income groups.

**Results:**

The anchoring vignettes exposed differences in scale responses across demographic groups. After adjusting for these biases, public transport users (OR = 0.37, 95 % CI 0.21–0.65), walkers (OR = 0.44, 95 % CI 0.24–0.82), and motor vehicle users (OR = 0.47, 95 % CI 0.25–0.86) were all found to have lower odds of reporting high QoL compared with bicycle commuters. Similarly, the odds of reporting high health satisfaction were found to be proportionally lower amongst all competing travel modes: motor vehicle users (OR = 0.31, 95 % CI 0.18–0.56), public transport users (OR = 0.34, 95 % CI 0.20–0.57), and walkers (OR = 0.35, 95 % CI 0.20–0.64) when compared with cyclists. Fewer differences were observed in the unadjusted models.

**Conclusion:**

Application of the vignettes by the two approaches removed scaling biases, thereby improving the accuracy of the analyses of the associations between travel mode and quality of life. The adjusted results revealed higher quality of life in bicycle commuters compared with all other travel mode users.

## Introduction

Subjective quality of life (QoL) is an important and widely used measure of health [1]. Quality-of-life assessments generally require respondents to rate their physical or psychological health status, or overall life satisfaction, on an ordinal Likert scale from ‘poor’ or ‘very poor’ to ‘very good’ or ‘excellent’. Single items or overall measures can be very useful indicators of health and health inequalities [2, 3]. Additionally, the brevity of single-item measures can reduce survey respondent burden and costs [3]. They are however prone to greater measurement error, which, if overlooked, may lead to inaccurate assumptions and conclusions.

Self-assessed scale measures can fail to provide meaningful results when there are differences in reporting behaviours across populations. Depending on their experiences and expectations, individuals interpret and respond to scale categories in different ways. Regardless of their underlying state of being, some people have a tendency to respond in the affirmative rather than to disagree, while others have a tendency to use the extreme or middle points of a scale. When this behaviour is systematic across population groups, it can lead to distorted or biased research findings. A number of terms have been used to describe these differences in scaling behaviour including ‘scale of reference bias’ [4], ‘response category cut-point shift’ [5], ‘reporting heterogeneity’ [6, 7], ‘differential item functioning’ [8, 9], and ‘scale perception bias’ [10].

In Western societies, people are generally positive about their overall QoL and will typically rate themselves towards the healthier end of a scale [11, 12]. However, differences in scale rating of QoL have been observed across age and gender, socio-economic, culture, and language groups [6, 12–14]. What makes subjective QoL so challenging to measure is that there is no universal agreement on how it is defined. As a result, many different instruments have been developed, each derived from a different conceptual understanding of QoL [15, 16]. Patient or survey respondents asked to rate their QoL may also interpret QoL differently, based on their own definition of QoL which is not necessarily in accord with definition presupposed by the researchers [17].

Given the importance of QoL as a health measure [1], disentangling reporting behaviour, incongruent interpretations of QoL, and population thresholds from latent well-being are essential for meaningful interpretation and comparison of subjective QoL data. The use of anchoring vignettes is one method for revealing scale perception bias and evaluating otherwise incomparable data. Vignettes are descriptions of hypothetical persons or situations that respondents are asked to rate on the same construct as a question about their own experience. The vignettes are rated on the same scale as the self-rated question [18]. The vignettes act as a set of reference points which are used to expose individual thresholds on a common scale. This allows the individual’s self-assessed responses to be assessed on the same dimension.

To date, few studies have used anchoring vignettes in the interpretation of QoL outcomes. Murray et al. [5] first applied vignettes to measure self-rated health across the WHO Multi-country Household Study on Health and Responsiveness. The methodology has since been applied to QoL measures including self-rated health and life satisfaction in only a few incidences, which is surprising given the large number of studies which have investigated QoL outcomes [19–25]. Often, researchers fail to investigate the presence of scale bias and provide biased results, or choose to remove the bias by discarding or analyse groups separately and avoid comparisons [26]. This is an unnecessary loss and can be avoided through application of the anchoring vignette approach.

It is possible that the low take-up of anchoring vignettes may be due to the perceived technicality of the anchoring vignette approach. Nonparametric rescaling of data and sophisticated multilevel regression modelling have been proposed as analysis methods [27, 28]. Nonparametric models recalibrate the distribution of responses to a comparable scale, by adjusting for the individual’s scale behaviour. In other words, the thresholds the individual used when they rated the hypothetical vignettes on a scale are then used to reinterpret and rescale the responses to a question about their own perceptions. The parametric models go further than simply rescaling the data by providing parameter estimates, and adjust for the variance of the individual thresholds in the scale responses. As both parametric and nonparametric methods have strengths and weaknesses, we apply both to compare QoL association with transport outcomes.

The Sydney Travel and Health Study (STAHS) is a longitudinal study of residents living in the inner-city suburbs of Sydney, Australia, which aims to measure the health (including QoL), transport, and economic impact of new cycling infrastructure [29]. How QoL is affected by changes in the urban built environment such as traffic and transport is an increasingly important issue in public health [30]. The detrimental effect of commuting stress on physical and psychological well-being is increasingly recognised [31, 32], while the benefits of more active modes of travel (primarily cycling and walking) are also gradually being understood [33, 34]. However, very few studies have sought to investigate QoL and transportation and compare differences between travel modes, specifically between active travel modes, and fewer still have included cycling. No transport and QoL study has as yet used anchoring vignettes and adjusted for scale perception bias.

With this in mind, the two primary purposes of this paper were to (1) examine scale perception bias in two single-item QoL questions: overall QoL and health satisfaction; and (2) model the relationship between commuting travel mode and QoL in the STAHS using nonparametric and parametric multilevel ordinal logistic regressions to adjust for these biases.

## Method

### Data sample

Cross-sectional baseline STAHS data were collected between September and October 2013 through an online survey. Respondents were recruited to the survey through multiple channels including random dial digit telephone calls to local residents, online panels, and community advertising. Consent was obtained as the respondent entered the survey web platform. Respondents were eligible if they lived within 5 km of the city centre (and exposed to a number of transport options), were aged 18–55 years, and had sufficient English to complete the survey. As part of a wider longitudinal study design, respondents had to have ridden a bicycle in their life and have no current disability preventing them from riding. A total sample of 846 responses was collected.

### Measures

#### Quality of life

QoL was measured using the abbreviated World Health Organization quality-of-life assessment (WHOQOL-BREF). Two umbrella items measured overall QoL and health satisfaction; ‘*How would you rate your quality of life?*’ (‘very poor’, ‘poor’, ‘neither good nor poor’, ‘good’, and ‘very good’) and *‘How satisfied are you with your health?*’ (‘very dissatisfied’, ‘dissatisfied’, ‘neither dissatisfied nor satisfied’, ‘satisfied’, and ‘very satisfied’). In addition, 24 items covered four specific domains: physical health, psychological health, and social and environmental facets of QoL. The WHOQOL-BREF was developed as a cross-cultural QoL instrument for use in the general population and has been validated in the Australian population [35].

#### Travel behaviour

To determine the association between QoL and commuting travel modes, participants were asked about their main mode of travel to work or study (public transport, motor vehicle, bicycle, or by foot). Bicycle travel was treated as the reference category.

#### Demographic and socio-economic factors

Demographic correlates with potential variation in reporting behaviour included sex and age (continuous). Education was dichotomised into tertiary or less than tertiary level. Annual household income was categorised in intervals from less than $20,000 to over $140,000 and dichotomised at AU$80,000+ or less [36]. Variables were dichotomised because of concerns about multiple categories reducing statistical power.

#### Anchoring vignettes

**Fig. 1:**
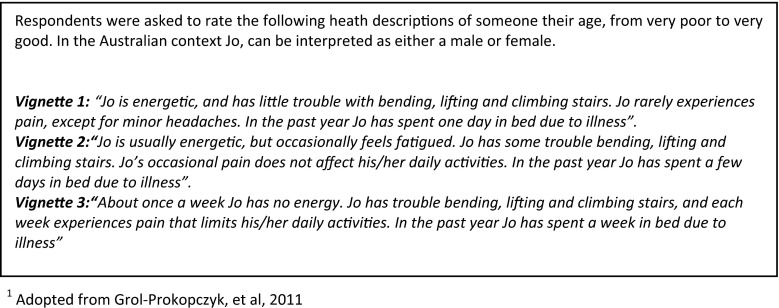
Health-related quality-of-life anchoring vignettes

A series of three vignettes were included to detect variations in QoL rating due to scale perception bias. The vignettes were of varying levels of general health of a hypothetical person called ‘Jo’, who respondents were to assume was of the same age as them (Fig. 1). Respondents were asked to rate the health status of Jo in each of the three scenarios. The survey then asked respondents to rate their own health and overall QoL using the same response scales. The way the respondents rated the three vignettes was then used to determine the thresholds they had applied to the self-rated question. The vignettes were based on Grol-Prokopczyk et al. [19]. The most severe scenario used by Grol-Prokopczyk and colleagues produced a floor effect in their healthy general population and was omitted from this current study. Unlike the study by Grol-Prokopczyk et al., in the present study, the vignettes were applied to the whole sample for nonparametric analysis. We hypothesised that while overall QoL and health satisfaction responses would differ, the same reporting behaviour was likely to exist across both QoL variables and that while overall QoL is broader than the physical health dimension, it would closely align.

The application of anchoring vignettes relies on two assumptions [8]. The first is the assumption of response consistency, that is, individuals will use the vignette response categories in the same way as they would when rating their own QoL. The second assumption is that of vignette equivalence, which requires that all respondents comprehend the vignette in the same way. In the case of these vignettes, vignette 1 should be understood by all respondents as a better level of health than vignette 2, followed by vignette 3. Any inconsistency in the rank order violates this assumption. There is however two different ways a response would be considered inconsistent. If someone rated the worst level of health [vignette 3] better than the other two vignettes, this shows that the respondent did not understand, or chose not to understand the question. However, some inconsistencies may occur due to the respondent genuinely perceiving two vignettes to be of the same level. These tied responses were included as appropriate, given the possibility that health states may be considered ‘equally good’ or ‘equally poor’, and provided they were otherwise consistent in rank order.

The vignettes were piloted to test transferability to an Australian population and confirm comprehension and face validity (*n* = 38). The vignettes performed as expected with respondents correctly ordering vignettes 1–3. No respondent misunderstood the intended order. Two respondents perceived V1 and V2 to be the same level of health, and one respondent perceived V2 and V3 to be the same level of health.

## Analysis

Data analysis was conducted as follows: data assumptions were tested; differences in reporting behaviours were then investigated; and then associations between QoL and transport modes were modelled using the two corrected approaches and compared with standard ordinal logistic regression analysis.

The distributions of the QoL and vignette variables were examined. The two lowest QoL categories (i.e. very poor and poor) were collapsed. The correlation between overall QoL and health satisfaction and WHOQOL physical health domain variables was tested (Spearman’s rho). The underlying assumptions of the vignettes were then evaluated. Lacking an objective measure of QoL, we investigated consistency across the three vignettes within the intended order. We also hypothesised that self-reported responses would be more likely to positively correlate with vignette 1 than vignette 3, and tested these correlations. We then tested the vignette equivalence according to the pattern where V1 ≥ V2 ≥ V3 and removed cases where this order was violated.

To illustrate scale perception bias, the rating of each vignette was compared between demographic groups (*χ*^2^). As the vignettes are fixed levels, there should be no difference between groups. For example, both men and women should rate the vignette in the same way. Significant associations (*p* < 0.05) would suggest different reporting behaviour between demographic groups. Income and education variables were also tested in their un-collapsed categories. The interaction between the QoL and demographic variables was then modelled using ordinal logistic regression.

Finally, the association between QoL and transport modes was analysed in three ways. A standard ordinal regression model was constructed, which adjusted for age, sex, income, and education. We called this the unadjusted model to differentiate it from the models correcting for scale perception bias. Secondly, scale biases were then corrected using the nonparametric approach described by King and Wand [27]. The QoL variables (overall QoL, health satisfaction) were rescaled according to the thresholds used by the respondent to rate the vignettes. The new QoL variables contained seven categories (based on the number of vignettes 2V + 1). If the self-rated response *X* was greater than the levels described by the vignettes, such that *X* > V1 > V2 > V3, then the new self-response Q was designated the highest category, seven and so forth (see Table 1 for full details). Where vignettes ratings were tied, for example *X* > V1 > V2 = V3, where V2 and V3 were given equal weighting, then more than one category would be valid. To deal with these inconsistencies, tied responses were designated the mean category of all possible categories that would apply for the given response. Inconsistent responses which violated vignette assumptions were excluded (*n* = 12). The rescaled variable was then analysed in the same way as the standard model.

**Table 1:** Nonparametric rescaling of quality-of-life (QoL) variables through the use of anchoring vignettes

Observed order	Consistent with expected order	New variable Q possible responses
*X* > V1 > V2 > V3	Ordered	7
*X* = V1 > V2 > V3	Ordered	6
V1 > *X* > V2 > V3	Ordered	5
V1 > *X* = V2 > V3	Ordered	4
V1 > V2 > *X* > V3	Ordered	3
V1 > V2 > *X* = V3	Ordered	2
V1 > V2 > V3 > *X*	Ordered	1
*X* > V1 > V2 = V3	Tied	7
*X* > V1 = V2 = V3	Tied	7
*X* > V1 = V2 > V3	Tied	7
*X* = V1 > V2 = V3	Tied	6
*X* = V1 = V2 > V3	Tied	3, 4, 5, 6
*X* = V1 = V2 = V3	Tied	2, 3, 4, 5, 6
V1 > *X* > V2 = V3	Tied	3, 4, 5
V1 > *X* = V2 = V3	Tied	2, 3, 4
V1 = V2 > *X* > V3	Tied	3
V1 = V2 > *X* = V3	Tied	2
V1 = V2 > V3 > *X*	Tied	1
V1 = V2 = V3 > *X*	Tied	1
V1 > V2 = V3 > *X*	Tied	1

Vignette responses are used to determine individual thresholds. Rescaling of the QoL variables creates a new variable, free from scale bias caused by differences in rating behaviour

In the final parametric model, the observed QoL response was allowed to vary according to the thresholds the respondent used, and individual thresholds are treated as a function of the covariates (as determined by the vignette anchor points). We first applied a hierarchical ordinal probit model in Stata using the gllamm function according to the example provided by Rabe-Hesketh and Skrondal [37]. We then applied a cumulative logit link. Logit models are more useful in explaining health outcomes and, unlike probit models, can be interpreted with odds ratios. The models’ fit was then compared using Akaike information criteria (AIC) [38] and Bayesian information criteria (BIC) [39], where the smallest criterion represents the model with the smallest information loss. As the models were non-nested and the complex design of the parametric model relied on transformed data, differentiating it from the previous models, the criterion information was weighted to the sample to reduce penalising the parametric model [40].

In each model, linearity of age was tested and confirmed as appropriate. Interaction terms were tested and effect modification rejected. For each model, the proportional log odds assumptions for ordinal logistic regression were tested, and no violation was observed. For missing income data (9 %) it was assumed a full-time student, unemployed, welfare recipient, or homemaker was less likely to be in the high bracket income. Otherwise, missing demographic data (missing income *n* = 3; education *n* = 6) were excluded, and only unique data retained. All statistical analyses were conducted using Stata version 13 (StataCorp LP, College Station, TX).

## Results

The sample characteristics for the STAHS data are given in Table 1. In this sample of inner-city residents, the main mode of travel commuters take to work or study is by public transport (39.2 %) followed by motor vehicle (23.4 %), foot (19.9 %), and bicycle (13.3 %) (Table 2).

**Table 2:** Characteristics of the Sydney Travel and Health Study cohort, Australia, and differences in scale rating across three vignettes

	Persons (*n* = 846) *N* %		Vignette 1 *X* ^2^ *p*	Vignette 2 *X* ^2^ *p*	Vignette 3 *X* ^2^ *p*
Sex					
Male	352	41.6	0.001	0.001	0.4
Female	494	58.4			
Age					
Mean (SD)	37.2 (11.1)				
18–34 years	363	42.9	0.5	0.2	0.02
35–55 years	483	57.1			
Income					
Less than $80,000	336	39.9	0.7	0.5	0.08
$80,000 or more	506	60.1			
Education					
Less than tertiary	255	30.4	0.9	0.7	0.2
Tertiary education	585	69.6			
Main mode of travel to work or study					
Public transit	332	39.2			
Car	198	23.4			
Walk	168	19.9			
Bicycle	113	13.4			
No travel	35	4.1			

Differences in the way demographic groups rated each vignette are presented in the right hand columns. A significant association (*p* < 0.05) indicates that demographic groups are rating the fixed vignettes differently

### Quality of life and vignette validity

The mean (SD) and distribution of responses for overall QoL and health satisfaction are given in Table 3. Overall QoL was skewed heavily towards the higher thresholds, while health satisfaction was more normally distributed broadly in line with a priori expectations [41]. The correlation between overall QoL and self-rated health satisfaction (rs = 0.55) and physical health (rs = 0.51) was satisfactory.

**Table 3:**
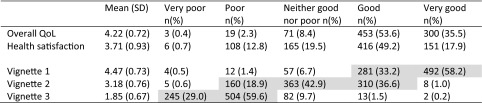
Distribution of QoL responses to anchoring vignettes in a sample of residents in Sydney, Australia (*n* = 846)

Shaded cells indicate weighting of vignette responses across upper and lower categories is in accordance with the level of health each vignette represents

The distribution of responses across response categories and mean values of the vignettes are also given in Table 3. As hypothesised, responses to vignette 1 were skewed towards the higher thresholds of the scale, while vignette 2 was distributed in the mid-point of the scale, and vignette 3 responses were skewed towards the lowest thresholds. The majority of responses (86 %) met vignette equivalence assumptions. Few respondents rated vignettes 1 and 2, or 2 and 3 on equal ranking (tied responses 12.6 %) and were retained. Only 1.4 % of vignette ratings was inconsistent and did not meet vignette equivalence, and these were removed from the analyses.

### Evidence of scale perception bias

In Table 2, the differences in reporting behaviour across the demographic groupings are presented for each vignette. If there was no scale bias, we would expect no association. The results suggest there is statistically significant difference in the way male and female respondents rated the higher health vignettes. No difference was observed between sexes in the way they rated the lowest level of health (vignette 3). This would suggest reporting differences on the higher end of the health continuum, where the majority of participants rate their QoL, and greater concordance between sexes on what is considered poorer health. Differences were also observed between how younger and older adults (binary age groups presented for illustration) rated the lower level of health. While respondents were asked to rate the vignettes based on someone their own age, this would suggest that the way different age groups rate poorer health differed. No reporting differences were observed according to income and education groupings.

### The association between travel mode and QoL

The relationship between commuting mode to work or study and quality of life is given in Table 4. All models also adjusted for the fixed effect of age, sex, income, and education. The standard unadjusted model suggests that public transport users were 2.08 times less likely to report high QoL than bicycle commuters (cumulative OR = 0.60, 95 % CI 0.39–0.93). In this model, no statistically significant differences were observed between cyclist and motor vehicle or walking mode users. However, after adjusting for scale response bias, motor vehicle users (0.47, 0.25–0.86), walkers (0.44, 0.24–0.82), and public transport users (0.37, 0.21–0.65), all had lower odds of reporting high QoL compared with bicycle commuters (parametric results).

**Table 4:**
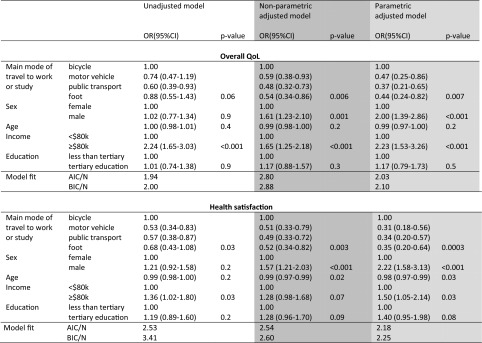
Ordinal logistic regression analysis of the association between QoL variables and commuting travel comparison between models unadjusted and adjusted for scale bias (*n* = 791)

Unadjusted and adjusted QoL modelled on cumulative proportional odds over the lower response categories. Excludes no mode of travel to work/study (*n* = 35)

Responses not confirming to vignette assumptions (*n* = 12) and missing socio-economic data (*n* = 8) are also excluded. Model fit information criteria are weighted to the sample dataset for comparison

In terms of health satisfaction, the odds of reporting a high health satisfaction in the unadjusted model were lower for motor vehicle and public transport users compared with bicycle commuters. After adjusting for scale perception bias, the odds of reporting high health satisfaction were found to be proportionally lower amongst all competing travel modes: public transport users (0.34, 0.20–0.57), motor vehicle users (0.31, 0.18–0.56), and walkers (0.35, 0.20–0.64) when compared with cyclists (parametric results).

### Comparison of regression models

The results of the rescaled nonparametric and the multilevel parametric regression analyses in Table 4 show similar findings despite some variations in the size of the coefficient and odds ratios (OR). Comparison of the loss of information in each model using the simplified weighted information criterions suggests a slightly better fit can be found in the parametric model over the transformed model in both the overall QoL and health satisfaction variables. The fit of the standard model while interesting to compare with the transformed variables is of course irrelevant if, as has been shown, the model is distorted by scale perception bias.

## Discussion

This study sought to adjust for the presence of scale perception bias in the self-rating of QoL in a sample of Australian city dwellers in order to appropriately analyse the relationship between commuting mode and QoL. Simple nonparametric rescaling of the data and parametric multilevel modelling was used to detect and adjust for differences in the rating behaviour across demographic groups. The vignettes were used to create fixed thresholds to compare findings. Application of the vignette methodology to the association between travel mode and QoL revealed some interesting findings that were not detected through conventional modelling. Using anchoring vignettes, we were able to detect significant differences in the overall QoL and health satisfaction between bicycle commuters and those who commuted by foot, motor vehicle, and public transport modes.

Demographic differences often exist across different modes of travel. For example, a higher proportion of men commute to work or study in Australia by bicycle or drive to work, while women are more likely to take public transport [42]. These mode share differences were reflected in this study. As a result of demographic differences in mode share, scale perception differences in QoL between demographic groups had a greater confounding effect on the relationship between travel mode and QoL than would have been observed had there been greater equality across travel modes.

To date, there has been very little research that has investigated the relationship between travel mode and well-being. Transportation appraisals and transport policy decisions too often fail to include the experience of the transport journeys from the user’s perspective with unconvincing efforts to translate subjective metrics of the user experience (comfort, convenience, QoL) into financial costs and benefits that can be compared alongside traditional measures such as travel time costs [43–45]. The association between transport QoL and health and well-being is however an emerging area of interest [45, 46]. The effect of travel on overall QoL and health has broader implications for infrastructure and urban planning and is particularly important in terms of sustainable transport investment. In many cities, such as Sydney, Australia, where these data were collected, commuting by bicycle is inhibited by a lack of cycling infrastructure and safe routes for travel. This has the potential to negatively impact on QoL. However, there is good evidence that moderately intense physical activity is associated with improved QoL and health satisfaction [47]. Cycling offers other benefits that may not be attained through other travel modes such as the mental health benefits of being outdoors, a greater control and predictability of the journey, sense of fun and excitement in the journey, and personal cost-savings [48, 49]. The higher intensity of cycling compared with walking may be what differentiates these modes in terms of QoL benefits. More research is needed to further explore causal associations between cycling and QoL.

The results of this study also provide a valuable illustration of the importance of measuring QoL appropriately. In the Canadian Community Health survey, Layes et al. [13] observed that health status consistently varied across age and socio-economic levels as a result of reporting behaviour. The authors concluded that ‘it might be misleading to take self-rated health at face value as a measure of health status’ [13]. For this QoL measure to continue to play an important role in population health research and policy development, they recommend that ‘its users must acknowledge and understand the determinants of self-rated health, including reporting behaviour’. QoL measures, particularly single items, face the problem of being undefined and therefore attract greater ambiguity. While there are many reasons why single-item QoL measures are used, we would argue that in order to make any comparison across individuals or populations, a common reference point needs to be introduced. The application of anchoring vignettes is one useful way of adjusting for reporting differences in scale threshold use, and of creating definitive parameters for abstract concepts such as QoL.

The standard ordinal logistic regression approach first used to analyse our data was unable to reveal actual associations due to scale biases. Logistic regression has been touted as an effective method for identifying reporting biases [26, 50]. Yet without some method to adjust for these scale biases, findings remain distorted. Two approaches were used in this study to adjust for scale bias, following those first proposed by King and colleagues [8, 51]. Parametric models provide greater precision over the nonparametric rescaling, yet they support the same outcome. One of the issues with the nonparametric approach is that any tied responses need to be scaled, and this becomes problematic when more than one of the scale categories are possible. However, there is a place for the more simplistic rescaled model over the decision not to adjust for scale bias. Parametric approaches require larger datasets and more sophisticated analysis. Nonparametric models which recalibrate the distribution of responses according to a common reporting scale are simpler to replicate and appropriate for less sophisticated statistical software, yet they require vignette questions to be asked of all respondents.

The QoL variables used in this analysis were taken from the two umbrella items in the WHOQOL-BREF. We tested the ability to use levels of health as vignette equivalences for health satisfaction and overall QoL in the assumption that scale perception bias for overall QoL could likewise be identified through the anchoring of responses to health specific scenarios. To confirm this, the correlation relationship between the single-item overall QoL and health satisfaction variables and health domains of the WHOQOL-BREF were tested.

The WHOQOL-BREF is designed for cross-country population use. While the content of the WHOQOL-BREF may be cross-culturally valid, differences in the interpretation of scales across populations are still likely to influence results, as observed in this study. The use of appropriate vignettes would address this limitation in the ability to compare findings across population groups.

The STAHS sample used in this analysis is a small sample of Australian inner-city residents. The sample was highly educated and as such not representative of the larger population. The sample was useful for this analysis because respondents were exposed to a number of public transport options and were included if they had ever ridden a bicycle. Thus, their choice of transport was not necessarily inhibited in ways other communities with lower access to transport options may be. This enabled us to investigate the association between QoL and a range of transport choices, their level of QoL may however be unrepresentative of the wider population.

## Conclusion

We found that anchoring vignettes were useful in detecting and correcting scale perception bias and reporting differences in two commonly used quality-of-life measures. Use of the vignettes improved the accuracy of the analyses and revealed important associations between travel mode and quality of life. After correcting for scale perception bias commuters who travelled by bicycle reporting higher quality-of-life scores than all other travel modes. Anchoring vignettes might be a powerful tool for improving the validity and interpersonal comparability of Likert-scale items in health research such as quality of life.
